# Cityscape LoRa Signal Propagation Predicted and Tested Using Real-World Building-Data Based O-FDTD Simulations and Experimental Characterization

**DOI:** 10.3390/s21082717

**Published:** 2021-04-12

**Authors:** Ricardo M. R. Adão, Eduardo Balvís, Alicia V. Carpentier, Humberto Michinel, Jana B. Nieder

**Affiliations:** 1 Ultrafast Bio- and Nanophotonics Group, INL—International Iberian Nanotechnology Laboratory, Av. Mestre José Veiga s/n, 4715-330 Braga, Portugal; ricardo.adao@inl.int; 2 Campus de Ourense s/n, Aerospace Engineering School, Universidade de Vigo, 32004 Ourense, Spain; 3 ERH-Illumnia, R. Feijoo 1, 32005 Ourense, Spain; ebalvis@gmail.com; 4 Defense University Center at the Spanish Naval Academy, University of Vigo, Plaza de España, S/N, 36920 Marín, Spain; avcarpentier@cud.uvigo.es

**Keywords:** LoRa, RSSI, RF propagation, O-FDTD simulations, Internet of Things (IoT), smart city

## Abstract

The age of the Internet of Things (IoT) and smart cities calls for low-power wireless communication networks, for which the Long-Range (LoRa) is a rising star. Efficient network engineering requires the accurate prediction of the Received Signal Strength Indicator (RSSI) spatial distribution. However, the most commonly used models either lack the physical accurateness, resolution, or versatility for cityscape real-world building distribution-based RSSI predictions. For this purpose, we apply the 2D electric field wave-propagation Oscillator Finite-Difference Time-Domain (O-FDTD) method, using the complex dielectric permittivity to model reflection and absorption effects by concrete walls and the receiver sensitivity as the threshold to obtain a simulated coverage area in a 600 × 600 m^2^ square. Further, we report a simple and low-cost method to experimentally determine the signal coverage area based on mapping communication response-time delays. The simulations show a strong building influence on the RSSI, compared against the Free-Space Path (FSPL) model. We obtain a spatial overlap of 84% between the O-FDTD simulated and experimental signal coverage maps. Our proof-of-concept approach is thoroughly discussed compared to previous works, outlining error sources and possible future improvements. O-FDTD is demonstrated to be most promising for both indoors and outdoors applications and presents a powerful tool for IoT and smart city planners.

## 1. Introduction and Related Work

The age of smart devices has arrived. With the increasingly powerful paradigm of the Internet of Things (IoT), smart city environments have become a pressing need for wireless technologies that enable efficient communication between distant objects [1,2]. Long Range (LoRa) [3] technology stands out for its combined low-power requirements and long-range coverage compared to other IoT communication protocols such as Bluetooth, Wi-Fi, ZigBee, or GSM. Such features are owed to its chirped spread spectrum and large bandwidth, making it less prone to interference and distance-fading. Examples of previous works deploying LoRa include monitoring systems for energy [4], environmental [5], and road condition monitoring [6], and flood prevention [7], among many other applications [8,9]. LoRa’s characteristics have enabled implementations using very low-power mobile [10] and even battery-free [6,11] devices. The main disadvantage of LoRa technology is the tradeoff between distance coverage and the data transfer rate. Strategies for tackling this limitation include LoRa variants such as the Carrier Sense Multiple Access (CSMA) mechanism, which has enabled long-range image transmission [12].

Unfortunately, a more extensive communication coverage range makes it difficult to accurately predict the signal strength within the covered area, which is crucial for an efficient network installation and hotspot deployment. Thus, understanding the propagation of LoRa signals is immensely relevant nowadays, at the birth of smart cities and in the future, when IoT-enriched technologies promise to prevail.

In this work, we predict the coverage map of a LoRa network with simulated Received Signal Strength Indicator (RSSI) maps obtained using a recently developed electric-field propagation method known as the Oscillator Finite-Difference Time-Domain (O-FDTD) [13]. We propose to use the dielectric permittivity of the absorbing buildings as a single parameter to model the presence of concrete walls, using real-world building distribution data extracted from Google Maps. In this manner, the low computational cost O-FDTD simulation method provides high-resolution cityscape LoRa RSSI maps. Also, we develop a simple experimental procedure to determine the coverage area based on monitoring the time of arrival of LoRa packets sent with a constant period by a moving transmitter. We use the area surrounding the university campus as the test environment. First, we compare the path-loss characteristics of the O-FDTD simulations with the conventional Free-Space Path Loss (FSPL) model. Then, we overlay the experimental and simulated coverage areas for comparison and calculate the area overlap. We obtain a good agreement between the predicted and characterized coverage area and find that the assumption of a single dielectric permittivity for all buildings within the analyzed area provides accurate mappings of the LoRa cityscape signal coverage, its local shadow regions, and hot spots.

## 2. Related Work

Previous works have experimentally analyzed the performance of LoRa systems for multiple parameters, with coverage areas ranging from typical indoors [14,15,16] to campus [17,18,19,20] and city scales [21,22,23,24]. Standard analytic prediction models are based on path-loss formulations optimized for specific power damping conditions, e.g., cityscapes [20,22] and others [25]. For the spatially-resolved RSSI, different numerical approaches may be considered, depending on the distance regime [22,26]: for shorter distances (line-of-sight), ray-based models are used [27,28]. For longer distances, diffraction effects become dominant, and either the uniform geometrical theory of diffraction (UTD) [29], often combined with ray-launching methods [30,31,32,33] (indoors), or knife-edge models [34,35] can be applied. Different knife-edge methods include the forward [36] and two-way [37,38] parabolic equation (PE)-based approaches, which are implemented either in the frequency [35] or time-domain [39,40]. For longer distances, scattering becomes the dominant effect, and the Okumura–Hata model [41] and the Longley–Rice irregular terrain model (ITM) [42,43] are most popular. The latter is prevalent because it uses real-world data, such as topography and weather parameters, to generate simulated RSSI maps. More recently, the updated ITM with obstructions model (ITWOM) [24,44] provides improved signal descriptions around obstacles.

In the cityscape regime, diffraction effects are dominant. This rules out the usage of simpler ray-based algorithms that do not take diffraction into account. Knife-edge methods such as the two-way PE [45] consider diffraction, but its application to complex real-world building geometries is challenging. The ITM is likely the most thorough method. However, it is highly demanding on computational requirements and simulation time. Recent efforts to improve the simulation times include a parallel graphical processing unit (GPU) [46] enhanced ITM implementations. Still, ITM is better suited for high power, very long-range scenarios, as ITM-generated maps usually lack the diffraction resolution required for city-scale low-power IoT and smart-city applications.

This work aims to overcome the lack of physical accuracy associated with standard ray-based signal-coverage models and single and two-way PE methods, which do not consider multiple reflections between building walls. We present a new approach to achieving high-resolution city-scale RSSI maps while minimizing the computational resources required. We propose using a versatile electromagnetic wave-propagation algorithm that considers crucial effects such as absorption and reflection. Such radiation-matter interaction algorithms are conventional in light-propagation applications, for which the Finite Element Method (FEM) [47] and the Finite-Difference Time-Domain (FDTD) [48,49] methods are the most popular. Both methods exist in commercially available software solutions. However, they focus on micro- and nano-scale light-matter interactions, resulting in computational requirements that are prohibitively large for city-scale spatial signal-propagation simulations. Alternatively, the recently developed O-FDTD [13] uses a much simpler approach than conventional FDTD. Instead of solving Maxwell’s equations for four field components, O-FDTD uses an approximation derived from the Lorentz Oscillator Model solution. The resulting formulation enables the field propagation using a single global electric field updating equation, which excludes the need for alternating orthogonal electromagnetic field components found in conventional FDTD. As a result, O-FDTD yields a single electric field component orthogonal to the 2D simulation space. The approach closely approximates the more complex electromagnetic FDTD predictions for dielectric materials and effectively reduces the memory and simulation time requirements, which enables its application to city-wide simulations. The method is implemented using Graphical User Interface (GUI)-power toolbox for MATLAB, making it both user-friendly and versatile, which is crucial for importing Google Maps data and comparing the simulation results against experimental data.

## 3. Materials and Methods

### 3.1. LoRa System Signal Coverage-Characterization

#### 3.1.1. Materials

**Figure 1:**
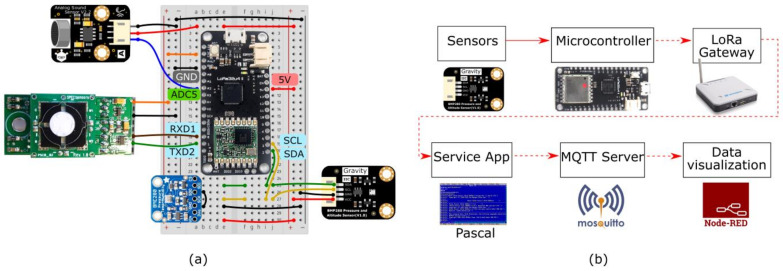
Implemented Long-Range (LoRa) Gateway system. (**a**) Schematic of the LoRa communication test device equipped with various sensors on an electronic breadboard. (**b**) Diagram of the communication process.

Figure 1 describes the implemented communication system, using several sensors for temperature (BMP280, DFRobot), humidity (BME280, Adafruit), noise/sound (Analog Sound Sensor v2.2, DFRobot), and NO_2_ concentration (DGS-NO2 968-043, Spec Sens) wired to a microcontroller board (LoRa32u4 II, BSFrance) (Figure 1a). The board features a LoRa node chip microcontroller (SX1276, AtmegaQR 32u4 3.3V @ 8MHz MCU), with transmitter power +20 dBm, receiver sensitivity between −136 dBm @ LoRa 125 Khz SF12 293 bps and −118 dBm @ LoRa 125 Khz SF6 9380 bps, and an operating frequency of 868 MHz and transmission current-consumption of 128 mA. A 5 V portable power bank drives the micro-controller circuit. The LoRa gateway (LPS8, Dragino), with a transmitter power of +20 dBm, was placed indoors (University of Vigo office), while the micro-controller LoRa-transceiver was taken outside for signal coverage measurements.

#### 3.1.2. Methods

The data packets are sent to a LoRa gateway using a simple custom-built LoRa packet format. The information that reaches the gateway is processed by a server service app that publishes the information in an MQTT server (running on a Raspberry Pi). The data is retrieved from the server, displayed on a Graphical User Interface (GUI), and stored in a file or database using Node-RED–see the schematic of the communication process in Figure 1b. The Node-RED’s GUI is made available in an online domain, making the measurements remotely accessible in real-time, e.g., via a smartphone (Galaxy S6, Samsung).

The microcontroller is programmed to send one data packet per second to the server for the signal-coverage measurements. Node-RED is used to display the received data and monitor the time elapsed between consecutive received packets. Since the signal time-of-flight is much shorter (picoseconds) than 1 s, one can assume that if the time interval between successive received packets is larger than 1 s, then one or more packets were lost. Hence, the time gap between received packets can be used to determine the coverage area by defining a maximum delay threshold and asserting that if the delay is below or above the threshold, then the communication is either established or broken, respectively. The transceiver location is tracked using the smartphone GPS and recorded using the Android app, Geo Tracker [50]. The mobile system (LoRa transceiver and smartphone) is then moved around the University campus in Ourense, while position-correlated time-between-packets measurements are collected and stored for analysis. The analysis consists of overlaying the reception rate with the GPS coordinates. The GPS coordinates are converted from angular to Cartesian using the Mercator projection:

(1) $$x(t)=\left(R_{0}+h\left(t\right)\right)\left(\theta \left(t\right)-\theta_{0}\right),$$

(2) $$y\left(t\right)=\;(R_{0}+h\left(t\right))\log\left[\tan\left(\frac{\pi }{4}+\frac{\phi \left(t\right)}{2}\right)\right]\;,$$

where *x*(*t*) and *y*(*t*) are the time-dependent Cartesian coordinates. *θ*(*t*), *φ*(*t*) are the time-dependent longitude, latitude, and altitude, respectively, in radians, and *h*(*t*) is the time-dependent altitude in meters. Table 1 shows the values used for the Greenwich meridian reference *θ*_0_ and Earth’s radius *R*_0_.

After obtaining time gaps between packet arrivals over the emitter trajectory, we use a simple interpolation method to create 2D maps of the time gap between packet arrivals. The developed process works similarly to a flood-fill algorithm, and in the following, we describe its use and function. First, a zero-filled 2D matrix of appropriate dimensions to represent the city map, e.g., 1 pixel per meter, is created, such that, e.g., a 700 × 700 m^2^ city map is represented by a 700 × 700 pixel matrix. The matrix cells (pixels) are then assigned to the measured time gap between packet arrivals following the relative GPS location data. Simultaneously, for those areas in which there are no measurements, the pixel entries remain zero. Optionally, it assigns the points beyond a certain (arbitrary) maximum radius with the maximum measured time gap between packet arrivals to prevent undesired outwards signal spreading. The resulting matrix is fed as starting point to the algorithm. The iterative algorithm assigns each zero-cell with the average of its non-zero closest neighbors. After each iteration, the map of the time gap between packet arrivals spreads 1 pixel in every direction, stemming from the original locations with the measured time gap between packet arrivals. The number of iterations required to fill the map depends on both the chosen matrix size and the number of points, and the spatial trajectory and shape. The MATLAB scripts used for data treatment and visualization and representative data files can be found in Supplementary Materials S1.

### 3.2. O-FDTD Simulation of LoRa Coverage

The cityscape LoRa propagation is simulated using the O-FDTD method [13] via the © 2020 WaveBox toolbox for MATLAB, Supplementary Materials S1. O-FDTD describes the propagation of electric field waves by modifying the Lorentz Oscillator Model (LOM). Similarly to the LOM, which considers the oscillation of atom-bound electrons driven by an incident electric field, O-FDTD uses a mesh of coupled oscillators whose orthogonal displacement relates to the electric field amplitude. However, O-FDTD’s oscillators are not electrons but abstract electric field carriers evenly distributed across space, including vacuum. Each oscillator is connected to its mesh neighbors by a refractive index-dependent coupling strength that defines the electric field transfer rate and, consequently, the wave propagation speed. The electric field *E* time propagation is obtained using the leap-frog backward differentiation method

(3) $$E_{i}=E_{i-1}+\frac{dE_{i-1}}{dt}\delta t+\frac{1}{2}\frac{d^{2}E_{i-1}}{dt^{2}}\delta t^{2},$$

where *t* is the time, *δt* is the temporal resolution, and the index *i* refers to the current time step. The single motion equation is formulated as:

(4) $$\frac{d^{2}E_{i-1}}{dt^{2}}=\frac{8}{3n^{2}\delta t^{2}}\left(E_{i-1}^{neigh}-E_{i-1\;}\right)-\frac{\sqrt{2}c}{n}\frac{4\pi \kappa }{\lambda }\frac{dE_{i-1}}{dt},$$

where *n* and κ are the real and imaginary parts of the refractive index, *E^neigh^* is the neighborhood electric field, and *c* and *λ* are the vacuum speed of light and wavelength. The first term in Equation (4) is analogous to the restoring spring motion as a function of the neighborhood electric field and accounts for the wave propagation and interference effects. The second term of Equation (4) relates to the energy damping or absorption. Using this simple formulation, O-FDTD allows simulating 2D maps of material-dependent electric field wave speed and absorption. The refractive index contrast induces signal reflections at the building walls.

The © 2020 WaveBox toolbox provides simple design functions and allows importing Google maps [51] data directly from a bitmap image. First, the map is converted into a vector format using Inkscape. Using Inkscape’s brightness-threshold bitmap-tracing function and minor manual editing, the city map is cleaned from features, such as text, icons, and roads, so that only building walls remain. Building walls are (arbitrarily) set as blue and empty space as black. We considered a 600 × 600 m^2^ map surrounded by 0.60 m margins used for the perfectly matched layers (PML) boundary conditions required by the O-FDTD algorithm and exported the final map as a bitmap *.png file. Air and the stone walls were modeled in WaveBox using refractive indices of *n*_air_ and *n*_wall_, respectively (deduced in Section 4.1). The simulation parameters are summarized in Table 2. For the sake of simplicity, the simulation can be carried out in the reverse of the experimental configuration, i.e., placing a dipole source at the experimental position of the LoRa gateway (receiver), located at the center of the simulation space. The O-FDTD simulation yields a 2D map of the signal power, which we converted into RSSI values given in dBm, representing the signal propagation across the cityscape. The coverage area is defined using the calculated (Section 4.1) receiver sensitivity as a threshold in the false color map. Detectable and undetectable values are shown in color and grayscales, respectively. The simulation of a 600 × 600 m^2^ area took about 3 h to complete. To visualize the simulation results described in Section 4.1, we overlayed a transparent vector-image of the city map onto the obtained RSSI values using Inkscape. 

## 4. Results

In this section, we present the LoRa coverage obtained via the conventional Free Space Path Loss (FSPL) method (Section 4.1) and compare it against the O-FDTD simulations (Section 3.2) that provide spatially-resolved LoRa RSSI and coverage maps in an urban environment. To demonstrate the accuracy of the O-FDTD simulations, we performed experiments on the university campus area and move the LoRa transceiver position while recording the time between packets per GPS position (Section 4.3). Ultimately, we quantified the differences between O-FDTD simulated and experimental signal coverage maps (Section 4.4).

### 4.1. Free Space Path Loss (FSPL) Model and Receiver Sensitivity

For this section, the LoRa network coverage was estimated using the RSSI predictions of the FSPL model. For this purpose, we determined the distance from the emitter at which the RSSI drops below the receiver’s decoding sensitivity. One can obtain more reliable signal path loss-based estimations for real-world applications by considering the detectability threshold approximately 10 dBm above the decoding sensitivity to account for the high packet losses for RSSI values close to the sensitivity limit. In the FSPL model, the attenuation of an electromagnetic signal can be expressed as:

(5) $$\mathrm{FSPL}={\left(4\pi fd/c\right)}^{2},$$

where *f*, *c*, and *d* are the signal frequency, the speed of light, and the distance from the emitter, respectively. The receiver sensitivity P_Rx_ quantifies the minimum RSSI that the receiver can decode and can be expressed as

(6) $$P_{\mathrm{Rx}}=\left(S/N\right)k_{0}T\left(\mathrm{BW}\right)F,$$

where S/N is the signal-to-noise ratio, k_b_ is Boltzmann’s constant, T is the temperature in Kelvin, BW is the signal bandwidth, F is the noise factor, and the product k_0_ × T × (BW) × F corresponds to the noise power.

Table 3 summarizes the signal parameters used in this work, whereby *NF* = 10log_10_(*F*) (dB) is the noise figure of the receiver architecture. Using Equation (6), we estimated the receiver sensitivity in dBm:

(7) $$P_{\mathrm{Rx}}=-7.5_{\left[\mathrm{dB}\right]}{+10\log\;}_{10}{\left(1{.38\times 10}^{-23}\times 290\times 1000\right)}_{\left[\mathrm{dBm}\right]}{+10\log}_{10}{\left(125,000\right)}_{\left[\mathrm{dB}\right]}{+6}_{\left[\mathrm{dB}\right]}=-124\;\mathrm{dBm},$$

which is in good agreement with reference values [54] and the product specifications. 

Using the FSPL model, we can express the free-space RSSI, P_FS_ as

(8) $$P_{\mathrm{FS}}{\;[\mathrm{dBm}]=G}_{\mathrm{Tx}}{+20\log}_{10}\left(\frac{c}{4\pi fd}\right).$$

**Figure 2:**
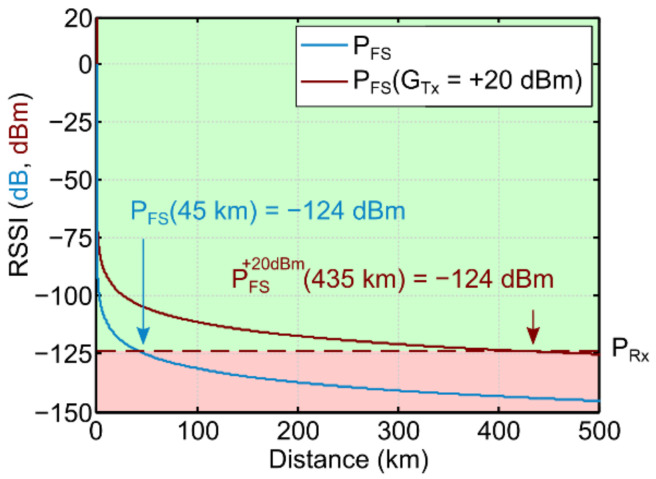
A plot of the calculated free-space Received Signal Strength Indicator (RSSI), P_FS_ power decay over distance, for an 868 MHz LoRa signal. The estimated receiver sensitivity P_Rx_ = −124 dBm yields maximum coverage distances of 45 km (blue) and 435 km (orange) for transmitter gains G_Tx_ of 0 dBm and +20 dBm, respectively.

Figure 2 shows free-space RSSI expressed in Equation (8) for two different transmitter gains *G*_Tx_ of 0 and +20 dBm. By finding the distance at which the RSSI drops below the sensor sensitivity, the maximum theoretical ranges of about 45 and 435 km can be estimated for *G*_Tx_ of 0 (1 mW) and +20 dBm (100 mW), respectively.

The relatively long coverage range achieved by LoRa networks is one of its main attractive features. However, no matter how impressing the free-space coverage ranges may be, they are hardly realistic in urban environments, where absorption, scattering, and interference effects induced by dense and intricate buildings can severely decrease the coverage range. 

**Figure 3:**
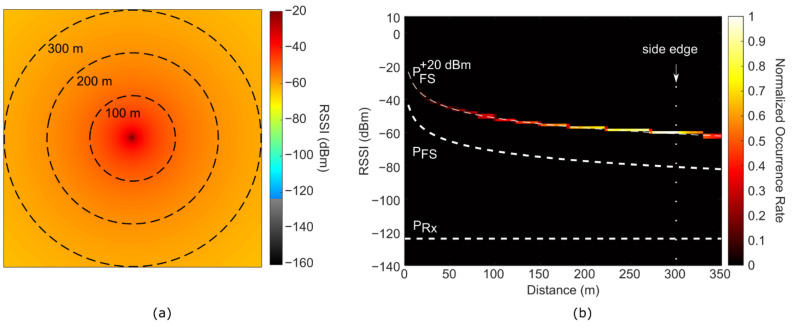
Theoretical free-space Received Signal Strength Indicator (RSSI) distribution of an 868 MHz LoRa signal over distance with a transmitter gain of G_Tx_ = +20 dBm. (**a**) 2D representation of free-space RSSI in a radius of 300 m. (**b**) 2D histogram of combined RSSI and distance occurrences in the RSSI map shown in (**a**). The color scale indicates the relative occurrence rate, normalized to the number of map pixels. The dashed white lines represent the free-space RSSI (P_FS_) for G_Tx_ of 0 and +20 dBm, and the receiver sensitivity P_Rx_ of −124 dBm, as indicated by the labels. The vertical dotted line indicates the 300 m spatial observation window used in the RSSI map representation in (**a**).

To simplify the comparison between free-space and cityscape scenarios, we plotted in Figure 3a a 2D representation of the RSSI (P_FS_) decaying from the central position. Figure 3b shows a 2D histogram of combined RSSI and distance occurrences in the theoretical P_FS_ map from Figure 3a. As expected, the 2D histogram shows a single line, which is in perfect overlap with *P*_FS_(*G*_Tx_ = +20 dBm). For additional reference, and similarly to Figure 2, we plotted the *P*_FS_(0 dBm) and receiver sensitivity, which shows that the free-space RSSI is far above the sensitivity threshold in these relatively short distances. It is worth noticing that the increase in occurrence rate (color gradient) for distances from 0 to 300 m is due to the larger circumference perimeter associated with longer radii. Furthermore, even though we show distances up to 350 m, the FSPL map is side-edge limited at a distance of 300 m from the center, which explains the decrease of the occurrence rate (dimmer color) for distances larger than 300 m. Nevertheless, the line continues to follow the P_FS_ dashed line.

In Section 4.2, we propose and test the use of the O-FDTD wave-propagation algorithm, which considers all the mentioned effects induced by the presence of buildings, for the realistic simulation of the propagation of LoRa signals in urban environments.

### 4.2. O-FDTD for Spatially Resolved LoRa Signal Propagation Simulation

The recently developed radiation-matter interaction O-FDTD algorithm [13], as implemented in the WaveBox toolbox for MATLAB, is used to simulate the propagation of LoRa signals in an urban environment, with building arrangements obtained from Google Maps. Here the O-FDTD method simulates the propagation of electric-field waves from an emitter (source) into a space characterized by different relative dielectric permittivity values, representing air *ε*_air_ = 1 or concrete buildings walls *ε*_wall_, respectively.

Previous reports have shown many different (even disparate) dielectric permittivity values for concrete [56,57]. The value considered in this work is derived as follows: We consider the real part of the optical refractive index n=2.5 for concrete [52], and, since many historic buildings in Ourense are stone-walled, knowing that a signal crossing a 60 cm stone wall is attenuated by about −30 dB [58], we calculated the absorption coefficient *α* = 0.115 dB/cm, using Beer–Lambert’s law

(9) $${P/P}_{0}{=e}^{-\alpha l},$$

where P/P_0_ is the power decay across a length *l*. The absorption coefficient as a function of the wavelength λ and the imaginary part of the refractive index *ñ* = *n* + i*κ* is expressed as

(10) $$\alpha =\frac{4\pi \kappa }{\lambda }.$$

The dielectric permittivity is simply the square of the refractive index

(11) $$\epsilon ={\left(n+i\kappa \right)}^{2}.$$

Thus, considering a wavelength of λ = 0.345 m (f = 868 MHz), using Equations (9)–(11) we estimated the dielectric permittivity of buildings walls *ε*_wall_ = 6.15 + i1.58 (*n*_wall_ = 2.5 + i0.316), which agrees with the average reported values [53].

**Figure 4:**
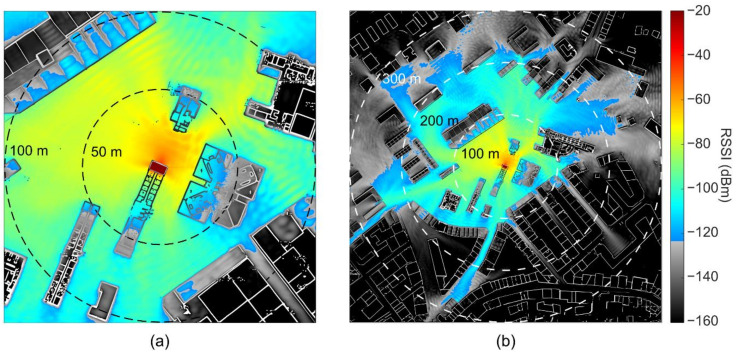
O-FDTD simulation of LoRa Received Signal Strength Indicator (RSSI) in Ourense for a signal source located indoors (center of the map). The building wall lines from Google Maps are overlaid onto the simulations for improved visualization. (**a**) Close-up 200 × 200 m^2^ map. (**b**) Zoom-out (full) 600 × 600 m^2^ map. The false color map turns to grayscale for RSSI values below the calculated receiver sensitivity of −124 dBm.

We performed the O-FDTD simulations for a portion of the city-map of Ourense, Spain, specifically, around the university campus, matching the realistic localization of the signal loss experimental characterization. Figure 4 displays the O-FDTD simulated RSSI maps. The emitter is located at the center of the simulation map, corresponding to the experimental position of the LoRa gateway. In the chosen false color map, the grayscale and colored areas identify the simulated RSSI values above and below the calculated *P*_RX_ = −124 dBm, respectively. In the close-up map Figure 4a, interesting signal heterogeneities such as local hotspots and wave-ripples are observed, emerging due to reflections from the buildings. Conversely, Figure 4b shows larger-scale effects such as ray-like reflections between buildings. The LoRa signal often appears trapped, displaying wave-guiding between buildings and beam divergence effects at the end of narrow streets. Despite the significant RSSI decrease induced by building walls (about 15 dBm/wall on average), the signal often finds its way around them, either by reflection or diffraction effects, especially in sparsely-built regions. On the other hand, shadow regions of reduced signal strength are observed in densely-built areas.

**Figure 5:**
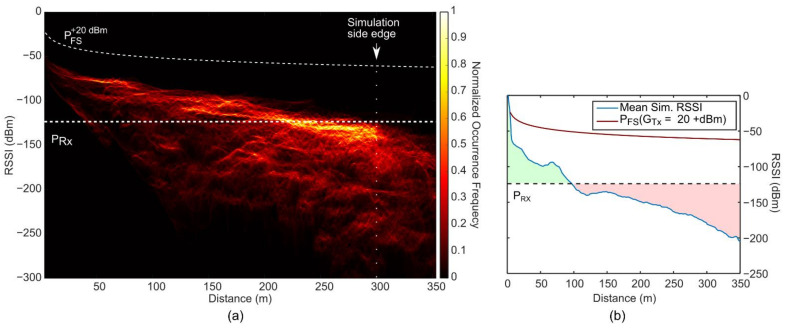
Statistical analysis of the real-world building-based O-FDTD simulated Received Signal Strength Indicator (RSSI). (**a**) 2D histogram of combined RSSI and distance occurrences in the simulated RSSI map. The color scale indicates the relative occurrence rate, normalized to the number of map pixels. The dashed white lines represent the free-space RSSI (*P*_FS_) for transmitter gains G_Tx_ of 0 and +20 dBm, and the receiver sensitivity P_Rx_ of −124 dBm, as indicated by the labels. The vertical dotted line indicates the 300 m spatial observation window used in the simulated RSSI map. (**b**) Free-space RSSI, P_FS_(G_Tx_ = +20 dBm) (orange) in comparison to the mean O-FDTD simulated RSSI over distance (blue). The green and red highlights indicate the distances for which the simulated RSSI is, on average, above and below the receiver sensitivity.

To compare the free-space decay scenario with the O-FDTD simulated, heterogeneity-rich cityscape scenario, we plotted in Figure 5a a 2D histogram of combined RSSI and distance occurrences together with the above-analyzed free-space model *P*_FS_(G_Tx_ = +20 dBm). The plot allows a statistical observation of the fraction of covered versus non-covered map locations that show RSSI values above (shorter distances) and below (longer distances) the receiver sensitivity. The overall much lower RSSI values than the free-space *P*_FS_(*G*_Tx_ = +20 dBm) indicate the rather drastic RSSI attenuation caused by the buildings. Figure 5b shows a plot of the average RSSI per distance. For distances up to 150 m, local minima and maxima can be assigned to the position of the first building layers, as both attenuate and concentrate the signal via wall absorption and reflections, respectively. It should be noted that the highly attenuated RSSI values inside the concrete walls themselves also contribute to the statistics, which leads to decreased average values in Figure 5b.

**Figure 6:**
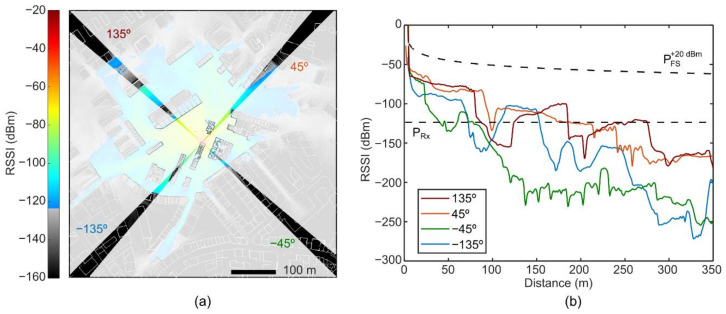
Distance dynamics of O-FDTD simulated Received Signal Strength Indicator (RSSI) for a sample of regions within the simulated cityscape maps. (**a**) Map of the arbitrarily selected propagation directions, with Δθ = 5° and θ = −135°, −45°, 45°^,^ and 135°. The vivid and faded-colored regions indicate the analyzed and disregarded areas. (**b**) Mean RSSI over distance for each of the selected propagation directions.

The statistical representations of Figure 5 characterize the overall dynamics of the full O-FDTD simulated RSSI map. The 2D histogram from Figure 5a builds upon the sum of all spatial contributions from propagation pathways stemming over 360° from the central source. Still, the RSSI decay characteristics for specific directions can be identified and analyzed in further detail. As an example, we apply an angular gating θ + ∆θ to analyze the RSSI over distance dynamics in four arbitrarily-defined directions. Figure 6a represents the selected gates of ∆θ= 5° and θ = −135°, −45°, 45° and 135°. The vivid and faded colors distinguish the considered from disregarded regions, respectively. Figure 6b shows the mean RSSI over distance in each direction of interest, where distinct direction-dependent dynamics with a strong dependence on the building density can be identified. In this case, the −135° and −45° directions show shorter coverage distances than the 45° and 135° directions. Interestingly, the RSSI drops in and out of the receiver sensitivity range, depending on the building arrangements.

According to the simulation results, and assuming a receiver sensitivity of −124 dBm, most of the simulated city area should lie within the LoRa network coverage. In Section 4.3, we present the experimental characterization of a LoRa network, and in Section 4.4, we compare the spatially resolved O-FDTD cityscape LoRa coverage simulations against the experimental results.

### 4.3. Experimental Characterization of LoRa Coverage

**Figure 7:**
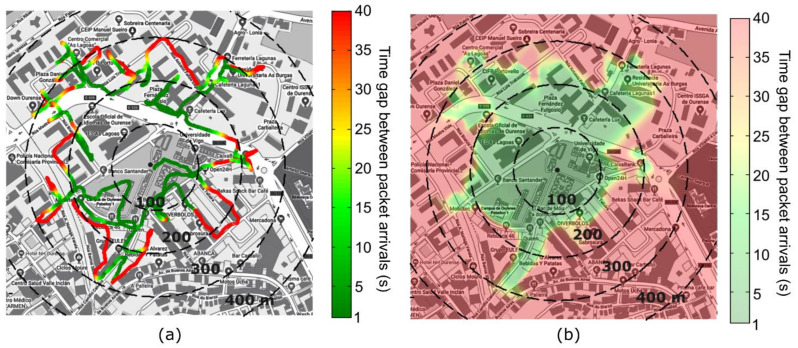
Mapping of time gap between packet arrivals around the LoRa gateway receiver. (**a**) Time gap between packet arrivals measured along the trajectory used to determine the edge of the signal coverage area. (**b**) Interpolated map of time gap between packet arrivals, obtained by expanding the results in (**a**) in all directions. The displayed city maps were retrieved from Google Maps [51].

This section presents the experimental results of the LoRa network coverage characterization in an urban environment. A mobile LoRa transmitter sends one LoRa packet per second containing multiple sensor measurements. The connection is tested by displacing the emitter and monitoring the timestamps of packet arrivals at the gateway as a function of the transceiver GPS position. We found that the time gap between packet arrivals changes directly from the set value of 1 s (connection established) and infinity (connection broken). Using this system, we determined the signal loss locations in every radial direction around the receiver by moving the transmitter around the gateway. Figure 7a shows a color-coded plot of the time gap between packet arrivals measured over the performed trajectory. The receiver is located at the center of the map. Using an iterative interpolation (flood-fill-like) script, we expanded the trajectory information (line) into a spread coverage area (2D color-coded map), as shown in Figure 7b.

**Figure 8:**
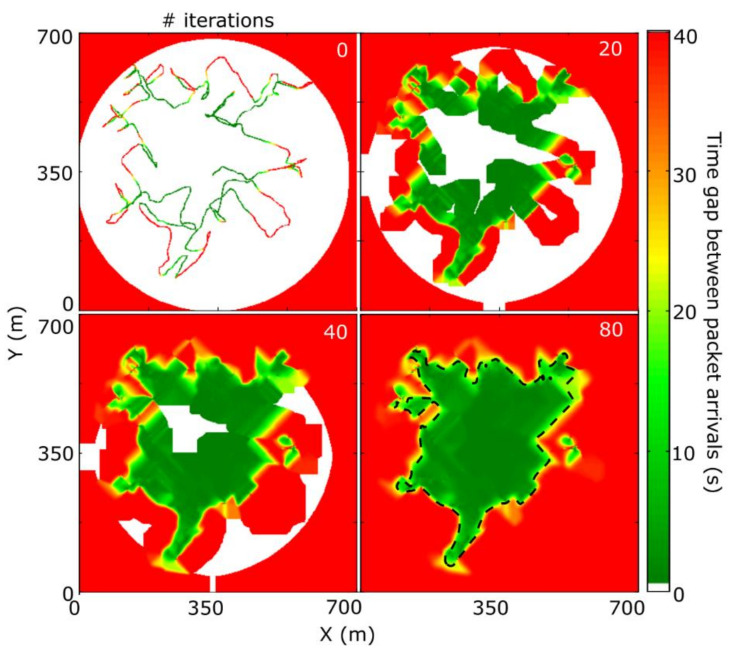
Expansion of experimental time gaps between packet arrivals measured along a line trajectory around the LoRa gateway into a map of time gap between packet arrivals, using an iterative flood-like interpolation algorithm. The four subplots show the results after 0 (starting point), 20, 40, and 80 iterations. The dashed line indicates the edge of the coverage area, defined as the interface where the time gap between packet arrivals equals 20 s.

The iterative progress of the integration algorithm is shown in Figure 8 for 0 (starting point), 20, 40, and 80 iterations. The resulting map shows two regions of time gap between packet arrivals equal to 1 s (network-covered areas) and “infinity” (here limited at 40 s, non-covered areas). The map shown in Figure 7b corresponds to the final result overlaid onto the city map. We defined the experimental coverage region, i.e., the region where the connection is established, as the locations for which the interpolated time gap between packet arrivals is lower than 20 s, whose edge is indicated by the dashed line in the final map of Figure 8.

In the following Section 4.4, the simulated and experimental coverage regions are overlaid and compared.

### 4.4. Analysis of Simulation Versus Experimental Results

**Figure 9:**
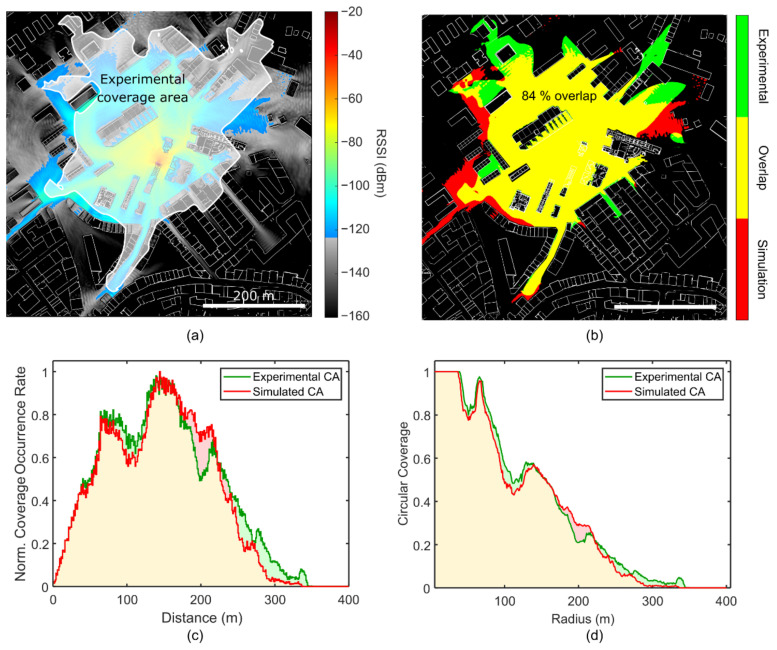
Correlation between simulation and experimental results. (**a**) Overlay of the experimentally determined coverage area onto the simulated Received Signal Strength Indicator (RSSI) map. (**b**) Color-coded overlay of (red) O-FDTD simulation only, (green) experimental only, and (yellow) overlapping coverage areas (CA). Spatial overlap of 84% between the simulated and experimental coverage areas is identified. (**c**) Histogram of covered distances and (**d**) circular coverage within the experimental (green) and simulated (red) coverage areas. The red, yellow, and green shades relate directly to the regions identified in (**b**).

Figure 9a shows the overlay of the experimental coverage area onto the O-FDTD simulation map. In Figure 9b, a color classification represents the match between simulated and experimental coverage regions. Matching regions are yellow, exclusively simulated are red, and exclusively experimental are green. The interpolation algorithm described above ignores the presence of buildings, which leads to inaccurate building edge descriptions. For a more accurate comparison, an adjusted/improved experimental coverage area was obtained by subtracting the building prints from the original experimental signal coverage map—see the difference between Figure 9a,b. To compare the coverage prediction with the experimental result, we calculated the total area overlap *η* as the ratio between the overlapping area *C_overlap_* and the sum of the simulation *C_sim_* and experimental *C_exp_* spatial coverage areas as follows:

(12) $$\eta_{area}=\frac{2\sum C_{overlap}}{\sum C_{sim}+\sum C_{exp}}.$$

A total area overlap *η_area_* = 84% can be identified. For the statistical analysis of the signal coverage as a function of the gateway distance, we defined the histogram *H(r)* of distance values within the coverage map *C* as

(13) $$H\left(r\right)=\mathrm{Hist}\left(C,r\right)=\sum_{x,y}R\left(r\right)\;⊙\;C,$$

where Hist(*A*,*r*) is the histogram of r values within a matrix *A*, *R*(*r*) is the binary map of the circumference with radius across the *xy* space and ⊙ is the element-wise (Hadamard) product. Then, we defined the radial coverage *H*_r_(*r*) as the coverage fraction over each circumference of radius *r* by normalizing *H*(*r*) by the perimeter of the rim, $\sum_{x,y}R\left(r\right)$

(14) $$H_{R}\left(r\right)=\frac{\sum_{x,y}R\left(r\right)⊙C}{\sum_{x,y}R\left(r\right)}=\frac{H\left(r\right)}{\sum_{x,y}R\left(r\right)}$$

The distance histogram *H*(*r*) and the circular coverage *H*_R_(*r*) are plotted in Figure 9c,d, for the experimental (green) and the simulated (red) coverage maps. The red and green shades identify the differences between the profiles and are directly related to the mismatching areas identified in Figure 9b. An average of 95 m of radial coverage range is obtained, which agrees with the transition point in Figure 5b, where the mean RSSI drops below the receiver sensitivity. A maximum LoRa signal coverage distance of about 340 m can be obtained from both approaches.

**Figure 10:**
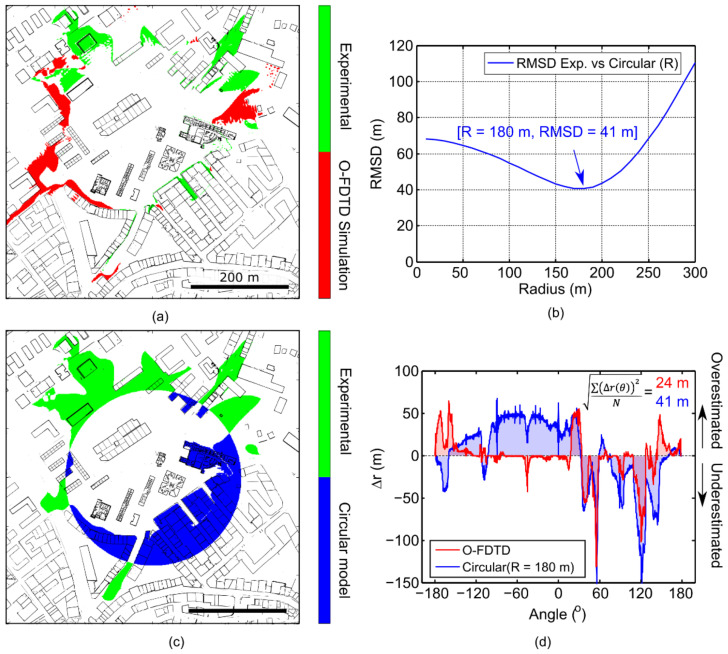
Correlation between the edges of the simulation and experimental coverage areas. (**a**) Color-coded overlay of O-FDTD simulation (red), experimental (green) coverage differences. (**b**) Minimization of the Root Mean Squared Differences (RMSD) between the experimental coverage area and a path-loss circular model. (**c**) Color-coded overlay of optimized circular model (blue), experimental (green) coverage differences. (**d**) Deviations of the O-FDTD (red) and optimized circle (blue) models from the experimentally determined coverage in dependence of the detection angle, with a 0.2 o resolution. Effective RMSD of 24 and 41 m are determined, respectively.

We analyzed the agreement between simulations and experiments at the edge of the determined signal coverage areas in more detail, see Figure 10. Figure 10a represents the differences between the experimental (green) and O-FDTD simulations (red). We aimed to compare with a simplified path-loss model, for which we allowed the coverage radius to fit the experimental values optimally (see Figure 10b). Projecting radius-dependent deviations over the detection angle ∆*r*(θ), the Root Mean Squared Deviation (RMSD) can be defined as

(15) $$\mathrm{RMSD}=\sqrt{\frac{\sum {\left(\Delta r\left(\theta \right)\right)}^{2}\;}{N}}$$

and be used as a quantitative descriptor of the coverage prediction quality, where

(16) $$\Delta r\left(\theta \right)=\int \left(C_{sim}^{\prime }\left(\theta \right)-C_{exp}^{\prime }\left(\theta \right)\right)dr$$

N = 360°/d*θ* is the number of angular points. We chose an angular resolution of d*θ* = 0.2°. We found that a radius of 180 m leads to the minimal RMSD of around 41 m for the path-loss circular model. Similarly, we plotted the spatial-resolved differences between prediction and experimental results in Figure 10c. Experimentally, we found an increased signal coverage towards North-West directions, exceeding the path-loss model predictions, while towards the South-East directions, the path-loss model overestimates the signal coverage, not being able to take the presence of a dense building arrangement into account. Figure 10d shows the angle-dependent deviations *δr*(*θ*) for the optimized circle and O-FDTD simulations. These deviations result in a net RMSD of 41 m, while the Google Maps building arrangement-supported O-FDTD simulations achieve a net RMSD value of 24 m, thereby showing the strength of the proposed model.

## 5. Discussion

This paper’s modeling and simulation results show the contrast between the simplest free space and a more realistic LoRa signal spatial distribution in a real-world cityscape. Despite its simplicity, the free-space path loss model Equation (8) provides some basic understanding of the propagating electromagnetic signals, specifically, the influence of frequency and transmitter gain in the signal strength attenuation over distance. The accurate prediction of the coverage range also depends on a correct receiver sensitivity calculation. In this work, the estimated value of −124 dBm was obtained partly from actual implemented LoRa parameters and reference values for the signal-to-noise ratio and noise factor (Table 3), which introduces some uncertainty. The FSPL model predicts a maximum free-space coverage range of up to 435 km for the parameters considered. Indeed, previous works (using different implementations) have set the experimental world record at an astonishing 766 km [59]. Other works have reported ranges of 9.7, 6.9, and 4.6 km in rural, urban and suburban environments [60], respectively. Also 1.6 km [23] and 1.2 km [19] were reported in urban environments, and reaching down to about 100 m in high-density urban environments [19].

We show in Section 4.2 that the wave-propagation O-FDTD method can be used to simulate the propagation of LoRa signals in urban environments and generate high-resolution RSSI maps. The relative dielectric permittivity defines the propagation conditions in air and concrete building walls. The imaginary part of the permittivity describes the signal absorption by the walls, while the dielectric contrast at the air/wall interfaces leads to reflections. Further, O-FDTD provides exact modeling of wave-effects, such as diffraction, which is particularly relevant in urban environments and disregarded by many other models, such as analytical path loss models and line-of-sight ray-tracing [43]. Yet, at larger scales, the O-FDTD also reproduces ray-like reflections, as shown in Figure 4, which is vital for distance regimes where scattering starts to play a dominant role [22]. However, despite considering building wall arrangements, several factors are not considered, such as other scattering objects like cars and trees, variations of terrain, weather parameters, the possibility of interference, and the influence of RF noise. Also, the signal mapping is incomplete inside buildings, and variable construction materials are disregarded (a single permittivity). Nevertheless, it provides excellent coverage predictions with 84%. In the following, we address each of the identified error sources and discuss improvements possibilities:

(1) Implementation and impact of cars and trees: Apart from path loss models specifically developed to account for trees [25], which require knowledge of tree parameters such as spatial distribution, leaf area index, and trunk diameter, most approaches use semi-empirical methods to determine an average path loss characteristic of the city in question [20]. We do not do this in our approach but instead use the building walls arrangement of a real-world city map directly. We remark that trees are sparse in this particular area of the campus, and cars do not entirely block the signal propagation above 2 m from the ground. Nevertheless, future works beyond this proof-of-concept could, in areas containing denser distributions of such scattering objects, include a map of trees or additional scattering elements assigned to specific regions where cars or trees are known to be abundant. The accurate location of trees may, for example, be obtainable from satellite images from Google Maps using some dedicated image processing algorithm. Such additional elements would be treated in the O-FDTD method analogously to the way building walls were treated in this work.
(2) Absence of terrain and weather data: As a 2D wave propagation method, O-FDTD does not trivially take terrain information into account. Future study could be envisioned to investigate how a modified O-FDTD approach, e.g., via dielectric permittivity gradients, could model terrain features. However, the topography should not significantly impact the area considered in this work since this is usually only considered for far more extended regions [44]. Previous works describe the influence of weather parameters in LoRa propagation [61]. Again due to the area contemplated, relevant air temperature gradients across the considered cityscape can be disregarded. A more refined approach could take into account shades cast by tall buildings in sunny countries. However, additional information on the building height would be required, and the shade regions vary with the hour of the day. Thus, a linear correction to the RSSI maps could be considered to account for the average temperature, following previously identified dependences of the signal-coverage on weather parameters.
(3) Effect of RF noise: Previous works demonstrate that high-levels of RF noise can be found in urban environments [62]. However, LoRa’s modulation characteristics make it particularly invulnerable to noise interference [54], and thus, we neglected such effects in our approximation.
(4) Indoor LoRa signal distribution: the concrete walls can be considered the dominant source of damping and reflection, not only because concrete is a very absorbent material in itself but also because walls represent full floor-to-ceiling barriers, while furniture items, aside from impossible to determine, are only partial barriers, thus allowing most of the signal to diffract around them. On the one hand, we consider all building walls as made of concrete and therefore ignore far less absorbent glass windows. This is a reasonable approximation since concrete is the predominant building material. On the other hand, one could argue that the exaggerated wall absorption may somehow compensate for the absence of furniture, even though the scattering features would be slightly different.
(5) Use of a single permittivity value: At a glance, this could be a significant error source since considering a single permittivity may overlook highly scattering materials such as metals. However, we are interested in modeling radiation-matter interactions, mainly in terms of reflection and absorption by building walls. In the RF regime, the dielectric properties of metals are dominated by their electrical conductivity, leading to a virtually infinite permittivity, making them almost perfect mirrors. In this work, we used a dielectric permittivity value estimated to reproduce the absorption of about 50 dB/m. Hence, the associated contrast in the dielectric permittivity, or equivalent refractive index, leads to a reflectance $r={\left(n-1\right)}^{2}/{\left(n+1\right)}^{2}$ of about 20% for normal incidence, which is not a very good approximation for metallic surfaces. The O-FDTD method can reproduce the metallic mirror behavior via the use of arbitrarily large refractive index values e.g., r(*n* = 100) = 96%, r(*n* = 200) = 98%, which is similar to previously reported approaches using knife-edge methods [35]. However, to accurately incorporate the cityscape contributions of metals would require a much more complete representation of the cityscape, containing the mapping of metals within the building walls. Considering this level of detail might be feasible for small-scale indoor applications, but it is not practical in the cityscape scope. Instead, we assumed that walls are dominantly composed of concrete, with a common permittivity. Alternatively, one could incorporate the effects of metals within the wall structures by increasing the real part of the refractive index to induce a higher reflectance, which could approximate the average metal composition in building materials, provided that information is available.

We demonstrate here that combining the O-FDTD method with a simplistic cityscape to building walls information from Google Maps, and using a single permittivity value, excellently predicts the LoRa signal distribution and coverage regions with high spatial accuracy. Compared to previous approaches that do not take real-world building maps into account, the proposed O-FDTD approach provides a significant improvement. Conceivable benefactors include end-users aiming to implement a continuous LoRa network in a dedicated area or network users to access the local LoRa network distribution information. Many previous works have used much more general approximations, ignoring real-world data-based cityscape heterogeneities [14,16,20,22,33,63,64]. Neural networks have been used to increase the computational efficiency of UTD ray-launching [65] methods to integrated spatial information but are still mostly limited to indoor scenarios. Knife-edge and PE approaches are the closest related to O-FDTD. However, such methods are limited in the number of wave reflections [36,37] and thus usually limited to a few knife edges or simplified models [45,66]. Multiple knife-edge diffraction [35] solves this issue but is still limited to simple geometries, which renders it unsuited for complex real-world building arrangements. ITM succeeds in real-world terrain and weather-dependent solutions but is highly demanding on computational resources. The simulation spatial resolution obtained is usually low compared to the achieved in this study. Therefore, it is typically applied to high-power telecommunications extending over extensive areas [22,24,44], far too large for the O-FDTD method.

The spatial resolution required by O-FDTD for the wave propagation may become unsustainable for LoRa maps much larger than the 600 × 600 m^2^ maps shown in this paper, depending on the RAM resources available. In O-FDTD, the spatial resolution (simulation step size) must be at least four times smaller than the signal wavelength, limiting the maximum simulation step size. As a result, the field-of-view of an NxN pixel simulation map depends on the signal wavelength. A possible strategy to increase the spatial step size, and thus the simulation’s field-of-view, is to use the following approximation: According to the FSPL model, a wave propagating with wavelength 2λ will see its signal strength attenuated by a factor of four, compared to a signal with wavelength λ, i.e., (*FSPL*)(2λ) = (*FSPL*)(λ)/4. This relation can be generalized as

(17) $$\mathrm{FSPL}\left(n\lambda \right)=\left(\mathrm{FSPL}\right)\left(\lambda \right)/n^{2},$$

and thus,

(18) $$P_{\mathrm{FSPL}}{(n\lambda )\;[\mathrm{dBm}]=P}_{\mathrm{FSPL}}{(\lambda )+20\log}_{10}\left(n\right),$$

which states the relationship between the RSSI of signals with wavelengths λ and *n*λ. Hence, an approximated result can be obtained by performing the simulation with a longer wavelength *n*λ and using Equation (13) to convert the RSSI map back to the λ scenario, thereby increasing the field-of-view by a factor of *n*, using the same number of pixels. It should also be noted that, according to the Beer–Lambert law, Equation (9), a change in the wavelength requires an updated wall dielectric constant. However, this approximation may introduce errors associated with (a) poor building resolution or (b) incorrect wavelength-dependent diffraction properties.

The LoRa signal coverage region was experimentally determined by thresholding the time-between packet arrivals, which is not as rich as an experimental RSSI mapping. However, the developed method provides a simple approximation of the binary signal coverage area, which can be achieved directly by any LoRa communication system, regardless of its simplicity, or the accurate knowledge of the receiver sensitivity or transmission power. This is particularly important for system implementations where the RSSI measurements are not possible. The developed interpolation algorithm provides a fast and straightforward solution to convert the time gap between packet arrivals from a line path to an area mapping. The main disadvantage of this method is that it does not consider the presence of buildings, which leads to unrealistic building edge and interior representations. We addressed this problem by performing a manual correction step, seen as the difference between the white semi-transparent area in Figure 9a and the green/yellow regions in Figure 9b. Previous works reported similar interpolation methods applied to experimental RSSI measurements [67]. However, very few provide complete cityscape RSSI maps, resorting, instead, to a few point measurement comparisons against path loss models.

The experimentally obtained maximum coverage range of 340 m is in good agreement with previous studies in urban environments [19]. Several strategies could increase the coverage range, such as using a higher spread factor, placing the LoRa gateway outdoors, and raising the gateway vertically to reduce overall signal reflections. However, the experimental conditions used in this work privilege such interactions and thus highlight the relevance of the O-FDTD simulations for low power, mid-range communications. Applications such as indoor, campus, or neighborhood-wide networks may benefit from smaller coverage areas, e.g., to reduce the chance of malicious communication interceptions.

As mentioned in Section 4.1, a reliable coverage range prediction (especially if based on simple path-loss equations) for a real-world application should consider the coverage threshold about 10 dB above the receiver decoding sensitivity, lest the actual range should fall short of the expected. We chose not to do this since the highly detailed O-FDTD maps correlated directly with the decoder sensitivity provide a sharper approximation between the O-FDTD simulated RSSI maps and the experimentally-determined signal-loss regions. 

The overlay between the experimental and simulated coverage areas reveals an area overlaps of 84%. However, most coverage area-prediction models yield good predictions near the gateway, where the coverage can be assumed homogeneous. A prime feature of the O-FDTD approach is the realistic coverage edge prediction, which is a massive advantage compared to simpler path-loss models that only predict average circular coverage ranges. As demonstrated in Figure 10 by overlaying coverage deviations at the edge, even the best-fitting circular model does not take buildings into account, leading to a much larger net RMSD of 41 m, compared to a RMSD of 24 m achieved by the O-FDTD simulations. While we considered this an excellent agreement, several sources of error in the experimental and simulation approaches are discussed. Our simulations did not consider temperature effects, while high temperatures are known to reduce the coverage range [61,68]. Indeed, the measurements were performed on a sunny day at 32 °C (89.6 Fahrenheit), leading to several-degree temperature-variations between sun and shade areas. The lack of terrain information may also play a role in the observed mismatches, as the model does not capture out-of-plane signal propagation. This effect may be the case of the regions highlighted in red on the left side of Figure 9b, where the terrain is lower than at the center of the cityscape. Furthermore, the incomplete experimental characterization of the citywide time gap between packet arrivals causes the experimental signal distribution to be more reliant on the simple interpolation algorithm. As discussed above, this introduces errors at the building interfaces, which are particularly noticeable on the top part of the map in Figure 9a, associated with the green areas in Figure 9b. Also, more refined (and more complex) projection models could be used to convert the GPS coordinates from the spherical to the Cartesian plane, thus improving the overlap between the experimental trajectory and the downloaded city maps, which is not perfect.

Altogether, the simulation and experimental results show an excellent agreement for LoRa signal distribution in the real environment, which holds the promise for future O-FDTD applications using spatial information maps for indoor and outdoor scenarios.

## 6. Conclusions and Outlook

The LoRa mid-range radiofrequency communication coverage area was spatially mapped in an urban environment and observed to be highly dependent on the building arrangement of the city. In this work, we report using the wave-propagation O-FDTD method combined with Google Maps data to obtain high-resolution simulated RSSI maps. The method reproduces both diffraction and scattering radiation-matter interactions, characteristic of the selected LoRa frequency and distance regime. This is a significant improvement from previous models, which usually consider such effects independently, often either suited for much smaller or much larger distance ranges or not easily adaptable to take complex real-world building maps into account. Also, we report a low-cost method to experimentally determine the binary “on-off” signal coverage area by mapping the cityscape time gap between packets and introducing a connection-determining threshold. A spatial overlap of 84% was obtained between the simulated and experimental signal-coverage areas. Furthermore, the deviations at the coverage area margins were far reduced compared to the best-fitting circular area that an average path-loss range model could yield. The simulations hold the advantage of providing highly detailed RSSI maps that display building fingerprints such as signal shadowing or hot spot formations, which would nearly be impossible to access experimentally. Future application of the proposed technique beyond this proof-of-concept demonstration may include the comparison of experimentally measured against O-FDTD simulated quantitative RSSI maps.

We conclude that this O-FDTD with spatial mapping is a versatile approach from which a vast set of applications may benefit. We foresee the wide use of this methodology for shorter-range wireless networks, such as Wi-Fi, Zigbee, or Z-wave in closed environments, or well-known LoRa alternatives such as NB-IoT or Sigfox. For indoor applications, besides the selected building wall position, other parameters may be included, such as furniture objects featuring different materials modeled via dedicated dielectric permittivity values, thus further enriching this simulation tool’s predictive power. This would provide value to city planners, engineers involved in smart city developments and improving the end-user experience by assuring homogenous signal coverage of information communication technology (ICT) networks.

## Figures and Tables

**Table 1:** Reference values for Greenwich meridian longitude reference *θ*_0_ and Earth radius *R*_0_.

*θ*_0_ [rad]	*R*_0_ [m]
$1.71\times 10^{-4}$	$6.3781\times 10^{6}$

**Table 2:** Parameters used for the Oscillator Finite-Difference Time-Domain (O-FDTD) simulation. *G*_Tx_ and *λ* are the emission power and wavelength, respectively, dx is the simulation spatial resolution, *n*_air_ and *n*_wall_ are the air and building wall refractive indices, respectively.

*G*_Tx_ [dBm]	*λ* [m]	d*x* [m]	*n*_air_	*n*_wall_
+20	0.345	0.0862	1	2.5 + i0.316 *

* Deduced in Section 4.1 from key assumptions and reference values [52,53].

**Table 3:** LoRa network parameters, where f, BW, and SF are the modulation frequency, bandwidth, and spreading factor, respectively. S/N and NF are the signal-to-noise ratio and noise figure, respectively. G_Tx_ is the transmitter gain.

f	BW	SF	S/N	NF	G_Tx_
868 MHz	125 KHz	7	−7.5 dB ^1^	6 dB ^2^	+20 dBm

^1^ Typical value for SF7 [55]. ^2^ Typical value [54].

## Data Availability

Data is contained within the article or Supplementary Materials.

## Supplementary Materials

The following are available online at https://www.mdpi.com/article/10.3390/s21082717/s1: Computer program O-FDTD simulation toolbox wavebox.m, MATLAB scripts used for data treatment and visualization: importGPX, convertGPXtoXY, importSensor, correlateSensorPos, mapSpreadLine, and example.m script calling the functions and representative data files: example.gpx, temp.txt.
